# Omaveloxolone inhibits IL-1β-induced chondrocyte apoptosis through the Nrf2/ARE and NF-κB signalling pathways *in vitro* and attenuates osteoarthritis *in vivo*

**DOI:** 10.3389/fphar.2022.952950

**Published:** 2022-09-27

**Authors:** Zengxin Jiang, Guobin Qi, Wei Lu, Hao Wang, Defang Li, Weibin Chen, Lei Ding, Xiuying Yang, Hengfeng Yuan, Qingmin Zeng

**Affiliations:** ^1^ Department of Orthopaedics, Shanghai Jiaotong University Affiliated Sixth People’s Hospital, Shanghai, China; ^2^ Department of Orthopedic Surgery, Fudan University Jinshan Hospital, Shanghai, China; ^3^ Department of Orthopedic Surgery, Zhongshan Hospital, Fudan University, Shanghai, China; ^4^ Department of Orthopedic Surgery, Shanghai TCM-Integrated Hospital Shanghai University of TCM, Shanghai, China; ^5^ Department of Orthopedics, The Second Affiliated Hospital of Chongqing Medical University, Chongqing, China; ^6^ Department of Radiology, Fudan University Jinshan Hospital, Shanghai, China

**Keywords:** osteoarthritis, omaveloxolone, Nrf2, NF-κB, apoptosis

## Abstract

Osteoarthritis (OA) is a common degenerative joint disease. Effective drugs that can halt or decelerate osteoarthritis progression are still lacking. Omaveloxolone is a semisynthetic oleanane triterpenoid exerting antioxidative and anti-inflammatory effects. The present study aims to determine whether omaveloxolone has a therapeutic effect on OA. Chondrocytes were treated with interleukin (IL)-1β to establish an OA cell model *in vitro*. Indicators of cell viability, oxidative stress, inflammation, cell apoptosis and extracellular matrix (ECM) degradation were investigated. Proteins related to the Nuclear factor erythroid derived-2-related factor 2 (Nrf2)/antioxidant response element (ARE) and nuclear factor kappa-light-chain-enhancer of activated B cells (NF-κB) signalling pathways were assessed using Western blotting. A destabilized medial meniscus surgery-induced OA rat model was used *in vivo*. Gait analysis, microcomputed tomography analysis, and histopathological and immunohistochemical analyses were performed to determine the therapeutic effect of omaveloxolone on attenuating osteoarthritis *in vivo*. The results showed that omaveloxolone exerts antioxidative, anti-inflammatory, antiapoptotic and anti-ECM degradation effects via activation of the Nrf2/ARE signalling pathway and inhibition of the NF-κB signalling pathway in chondrocytes *in vitro* and attenuates OA progression *in vivo*, suggesting that omaveloxolone may be a potential therapeutic agent for OA.

## Introduction

Osteoarthritis (OA) is a chronic degenerative disease characterized by the degeneration of articular cartilage. The prevalence of OA has been rapidly increasing owing to an ageing population as well as an obesity epidemic. Approximately 300 million people suffer from OA worldwide (Vitaloni et al., 2020). OA is a leading cause of disability (Hunter et al., 2020). Nonsteroidal anti-inflammatory drugs (NSAIDs), selective cyclooxygenase-2 inhibitors and acetaminophen are used clinically for symptomatic relief. However, they cannot effectively prevent the progression of OA (Abdel-Aziz et al., 2021; Zhang et al., 2021b). Patients with severe OA eventually require arthroplasty. OA constitutes a considerable burden on society worldwide (Hawker and King, 2022).

Chondrocytes, the only cell population of articular cartilage, play a crucial role in maintaining articular cartilage homeostasis (Chen et al., 2021). Hence, the survival of chondrocytes is critical and essential for the structural preservation, functional regulation and extracellular matrix (ECM) turnover of articular cartilage (Hall, 2019). Growing evidence demonstrates an association between chondrocyte death and OA (Yang et al., 2021). Increased reactive oxygen species (ROS) generation and inflammation can initiate chondrocyte apoptosis, which contributes to the occurrence and development of OA. Inhibiting chondrocyte apoptosis is a potential therapeutic intervention for OA (Blanco et al., 1998; Hashimoto et al., 1998; Hwang and Kim, 2015; Chow and Chin, 2020).

Omaveloxolone (also known as RTA408) is a semisynthetic oleanane triterpenoid and a novel compound in the antioxidant inflammation modulator class (Probst et al., 2015). Omaveloxolone is one of the most potent activators of the nuclear factor erythroid derived-2-related factor 2 (Nrf2)/Nrf2 activates antioxidant response element (ARE) pathway (active at nanomolar concentrations) (Cuadrado et al., 2018). Increasing evidence indicates that targeting the Nrf2/ARE signalling pathway is of pharmacological interest in the treatment of OA. Nrf2 is a critical regulatory factor in the system of oxidative stress defence. Nrf2 binds to the inhibitory protein Kelch-like ECH-associated protein 1 (Keap1) in the cytoplasm under physiological conditions. Nrf2 dissociates from Keap1 and translocates into the nucleus under stress or pathological conditions. ARE-regulated antioxidant proteins, including haem oxygenase-1 (HO-1) and NADPH quinone oxidoreductase 1 (NQO-1), to exert antioxidant effects (Ulasov et al., 2021; Panda et al., 2022). Several previous studies have confirmed that the Nrf2/ARE signalling pathway plays a protective role in articular cartilage (Marchev et al., 2017; Song et al., 2021; Chen et al., 2022). Consequently, agents, including omaveloxolone, may have therapeutic potential for the treatment of OA for their ability to activate Nrf2/ARE signalling pathway.

In addition, omaveloxolone is also an effective inhibitor of the Nuclear factor kappa-light-chain-enhancer of activated B cells (NF-κB) signalling pathway (Sun et al., 2020; Zhang et al., 2021). NF-κB is also an important regulator of oxidative stress and inflammation. NF-κB dimers interact with inhibitory IκB proteins in the cytoplasm under physiological conditions. NF-κB dimers translocate from the cytoplasm into the nucleus to activate NF-κB-dependent genes following injury or stress. The canonical and noncanonical pathways are the two major signalling pathways involved in the activation of the NF-κB signalling pathway. The canonical pathway is dependent on IKKβ and NEMO, while the non-canonical pathway is dependent on IKKα(Rigoglou and Papavassiliou, 2013; Jimi et al., 2019). The NF-κB signalling pathway has been identified as a key contributing factor that is abnormally activated in OA. Inhibition of the NF-κB signalling pathway shows therapeutic potential in the treatment of OA (Marcu et al., 2010; Choi et al., 2019; Lepetsos et al., 2019). It is conceivable that omaveloxolone with a suppressive role in NF-κB signaling would have therapeutic potential for OA.

Omaveloxolone has been shown to have strong antioxidant and anti-inflammatory effects by activating the Nrf2 pathway and/or suppressing the NF-κB signalling pathway *in vitro* or *in vivo* in studies of some diseases, such as acute asthma exacerbation, nonalcoholic steatohepatitis, acute kidney injury and neurodegenerative diseases (Han et al., 2017; Yang et al., 2019; Zhang et al., 2019; Reisman et al., 2020). In addition, the safety of omaveloxolone was preliminarily confirmed in a phase Ⅰ clinical trial in patients with metastatic non-small-cell lung cancer or melanoma and a phase Ⅱ clinical trial in Friedreich ataxia (Creelan et al., 2017; Lynch et al., 2019; Madsen et al., 2020).

Given the effects of omaveloxolone on the regulation of the Nrf2/ARE and NF-κB signalling pathways and the critical role of the NF-κB pathways in OA, omaveloxolone may have potential as a therapy for OA. However, supporting evidence is still lacking. We thus conducted the current study to explore the roles and mechanism of omaveloxolone in protecting chondrocytes against IL-1β-induced cell apoptosis *in vitro* and to determine the potential therapeutic effects of omaveloxolone on preventing OA *in vivo*.

## Materials and methods

### Cell isolation and culture

All animal experiments in the present study were approved by the Committee on Ethics of Animal Experiments of Fudan University Jinshan Hospital (Shanghai, China). Four, 4-week-old male Sprague-Dawley (SD) rats (Shanghai SLAC Laboratory Animal Co. LTD) were euthanized by an overdose of carbon dioxide. The cartilage of the hip joints was harvested, cut into small pieces and digested with 0.1% collagenase II (Gibco; Grand Island, NY, United States) at 37°C overnight. The chondrocytes were collected and cultured in Dulbecco’s modified Eagle’s medium (DMEM, Gibco) with 10% foetal bovine serum (FBS, Gibco) in a 5% CO2 atmosphere at 37°C.

### Cell viability assay

The cell viability of chondrocytes was measured using a Cell Counting Kit-8 (CCK-8, Beyotime Institute of Biotechnology, Shanghai, China). Cells were treated with different concentrations of omaveloxolone (5, 10, 25, 50, 100, 250, 500, 1000 and 2000 nM) for 24, 48 and 72 h. IL-1β (10 ng/ml, PeproTech EC, London, United Kingdom) was used to mimic inflammatory conditions (Jin et al., 2021). DMEM containing 10% CCK-8 solution was added to each well for 2 h. Absorption was detected at 450 nm using a microplate reader (Epoch; BioTek Instruments Inc., Vermont, United States).

### ROS level evaluation

2′,7′-dichlorofluorescein diacetate (DCFH-DA, Sigma-Aldrich, Missouri, United States) staining was used to evaluate ROS levels in chondrocytes 24 h after treatment. Briefly, chondrocytes were washed with PBS twice, stained with 10 μM DCFH-DA for 30 min, washed with serum-free DMEM three times to remove the DCFH-DA solution and observed under an Olympus FV3000 confocal laser scanning microscope. In addition, cells were collected and stained with DCFH-DA for flow cytometry analysis using a BD Accuri C6 plus flow cytometer (BD Biosciences, Vianen, Netherlands) to quantify ROS levels.

### Malondialdehyde (MDA) and superoxide dismutase (SOD) evaluation

MDA content in chondrocytes was detected using an MDA assay kit (Beyotime). SOD levels in chondrocytes were determined using a SOD assay kit (Nanjing Jiancheng Bioengineering Institute, Jiangsu, China). In brief, cell lysates were cultured with working buffer for 30 min at 37°C according to the manufacturer’s instructions 24 h after treatment. Absorbance was detected at a wavelength of 523 nm (MDA) or 450 nm (SOD) using a microplate reader. A bicinchoninic acid (BCA) protein assay kit (Beyotime) was used to determine the total protein concentration to normalize the MDA and SOD levels.

### Mitochondrial membrane potential determination

The mitochondrial membrane potential of chondrocytes was detected using a JC-1 mitochondrial membrane potential assay kit (Abcam, Cambridge, United Kingdom) 24 h after treatment. Chondrocytes were stained with JC-1 (5 μg/ml) for 25 min followed by Hoechst 33,342 (Thermo Fisher, Waltham, MA, United States) staining for 5 min at 37°C. The cells were then observed under an Olympus FV3000 confocal laser scanning microscope. In addition, cells were collected and stained with JC-1 for flow cytometry analysis using a BD Accuri C6 plus flow cytometer to quantify mitochondrial membrane potential levels.

### Annexin V-fluorescein isothiocyanate (FITC)/propidium iodide (PI) staining

Chondrocyte apoptosis was measured using an Annexin V-FITC kit (BD Bioscience, CA, United States) 24 h after treatment. Then, 5 μL Annexin V was added to the chondrocytes for 30 min, and 5 μL PI was added for 5 min at 37°C in the dark. Flow cytometry analysis was performed using a BD Accuri C6 plus flow cytometer. Chondrocytes were observed under an Olympus FV3000 confocal laser scanning microscope.

### Western blotting

Chondrocytes were lysed in radioimmunoprecipitation assay buffer to extract total protein. In addition, a nuclear protein extraction kit (Beyotime) was used to extract nuclear protein from chondrocytes. Proteins were separated by sodium dodecyl sulfate-polyacrylamide gel electrophoresis and transferred to polyvinylidene fluoride membranes (Beyotime). Membranes were incubated overnight at 4°C with the following primary antibodies: inducible nitric oxide synthase (iNOS, 1:2,000, ab178945, Abcam), cyclooxygenase-2 (COX-2, 1:2,000, ab188183, Abcam), B cell lymphoma 2 (Bcl2, 1:2,000, ab196495, Abcam), Bcl2-Associated X (Bax, 1:2,000, ab32503, Abcam), metalloproteinase with matrix metalloproteinase (MMP) 3 (1:2,000, ab52915, Abcam),MMP13 (1:2,000, 18165-1-AP, Proteintech, Wuhan, China), collagen type II (1:2,000, ab188570, Abcam), aggrecan (1:2,000, 13880-1-AP, Proteintech), phosphorylated P65 (p-P65) (1:1,000; ab76302, Abcam), P65 (1:1,000; ab19870, Abcam), p-IκBα (1:1,000; ab133462, Abcam), IκBα (1:1,000; ab32518, Abcam), Nrf2 (1:1,000, 16396-1-AP, Proteintech), HO-1 (1:2,000, ab13243, Abcam), NQO-1 (1:2,000, 11451-1-AP, Proteintech), Lamin B1 (1:2,000, 12987-1-AP, Proteintech) and β-actin (1:5,000; #4970, Cell Signaling Technology Inc., Danvers, MA, United States). Membranes were subsequently cultured with the corresponding secondary antibodies (horseradish peroxidase-labelled goat anti-rabbit IgG and anti-mouse IgG, Proteintech). Signals of target proteins were visualized using enhanced chemiluminescence on an imaging system (Tanon, Shanghai, China). Relative protein levels were quantified using ImageJ software (version 1.8.0; National Institutes of Health, Bethesda, MA, United States) normalized to β-actin or Lamin B1.

### Immunofluorescence assay

Immunofluorescence was performed to explore the changes in the expression of aggrecan, collagen type II, MMP3 and MMP13. Chondrocytes were incubated in blocking solution and stained with primary antibodies against aggrecan, collagen type II, MMP3 and MMP13 overnight at 4°C. Chondrocytes were then stained with the FITC- or phycoerythrin (PE)-conjugated secondary antibody (Thermo Fisher). The nucleus was labelled with Hoechst 33,342. Cells were observed under an Olympus FV3000 confocal laser scanning microscope.

### Animal model

Twenty-four 4-week-old SD rats were used for *in vivo* experiments. Animal experiments were performed after 1week adaptive feeding. Destabilized medial meniscus (DMM) surgery was performed to induce OA in the right knee of rats as described previously (Lin et al., 2022; Teng et al., 2022). The rats were randomized to four groups (sham group, DMM group, DMM +200 μg/kg omaveloxolone group and DMM +500 μg/kg omaveloxolone group), with six rats in each group. Omaveloxolone was dissolved in normal saline. Rats received intraperitoneal injections of omaveloxolone every 3 days. Intraperitoneal injections of omaveloxolone were performed 2 weeks after modeling. The omaveloxolone dosages were based on our pre-experiment results and previous study (Sun et al., 2020).

### CatWalk XT gait analysis

The Catwalk-gait test was performed using the Catwalk automated gait analysis system (Noldus Information Technology, Wageningen, Netherlands). Rats walked freely on a glass floor lit by green light, and the position of the rat footprints was recorded by a high-speed video camera. The recorded data were analysed using catwalk program software (Noldus, CatWalk XT version 10.6.608).

### Radiographic analysis

Increasing evidence indicates that subchondral bone changes are crucial pathological changes in OA (Li et al., 2013; Aizah et al., 2021; Hu et al., 2021). Hence, structural alterations of subchondral bone architecture were evaluated in this study. Rats were euthanized by carbon dioxide 8 weeks after the omaveloxolone treatment. The right knee joints were harvested. Microcomputed tomography (micro-CT) images of the knee joints were obtained using a SCANCO 50 (Switzerland). Three-dimensional (3D) images of the knee joints were reconstructed. Bone morphometric parameters of tibial subchondral bone, including bone mineral density (BMD), bone volume (BV)/total volume (TV), trabecular number (Tb. N), trabecular separation (Tb. Sp) and trabecular thickness (Tb.th) were analysed.

### Histopathology and immunohistochemistry analysis

Knee joints of rats were fixed with 4% paraformaldehyde and decalcified in 10% ethylenediamine tetraacetic acid (EDTA). Tissues were processed and embedded in paraffin for histopathological examination. The sections were stained with haematoxylin-eosin (HE), safranin O/fast green (SO/FG) and toluidine blue (TB). For IHC analysis, antigen retrieval was performed using microwave treatment. Endogenous peroxidases were blocked using 3% hydrogen peroxide. Goat serum (10%) was used to block nonspecific staining. Sections were incubated with the primary antibody against collagen type II and aggrecan at 4°C overnight, followed by the secondary biotinylated antibody (Proteintech) for 30 min at 37°C. Sections were stained with 3,3′-diaminobenzidine tetrahydrochloride for 25 s and then stained with haematoxylin for 5 min at room temperature. Sections were digitally scanned using a BX51 Olympus fluorescence microscope. Collagen type II levels were quantified using ImageJ software.

### Statistical analysis

All experiments were replicated independently three times. Data are presented as the mean ± standard deviation. A two-tailed Student’s t test was applied for two-group comparisons. The Mann–Whitney test was used for nonparametric data (OARSI scoring and Mankin scoring). *p* values <0.05 were considered statistically significant. Statistical analyses were performed using SPSS software (version 22.0; IBM Corp.).

## Results

### Omaveloxolone improved the viability of chondrocytes exposed to IL-1β

**FIGURE 1:**
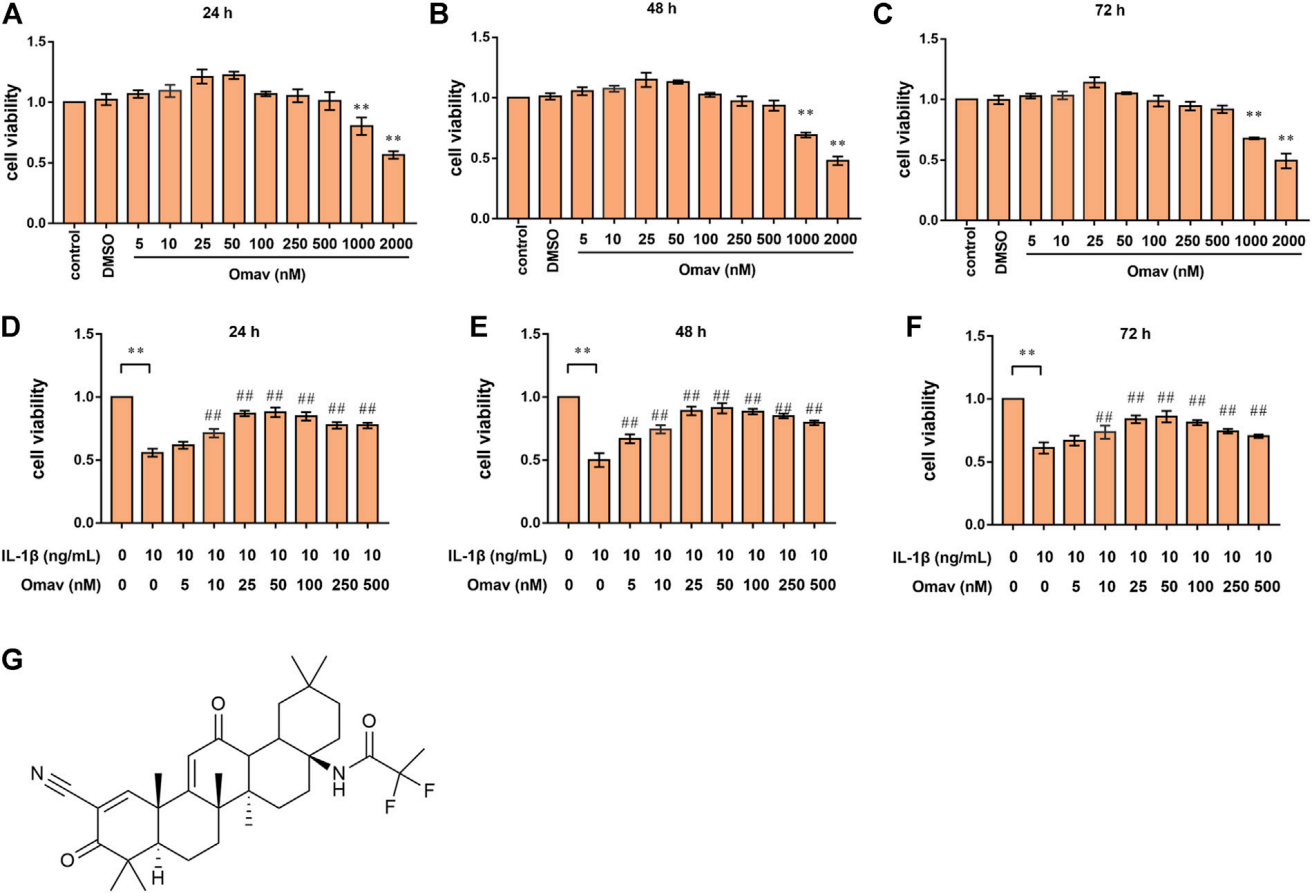
Omaveloxolone improved the viability of chondrocytes treated with IL-1β *in vitro*. Cell viability of chondrocytes treated with different concentrations of omaveloxolone (5, 10, 25, 50, 100, 250, 500, 1000, and 2000 nM) at 24 h **(A)**, 48 h **(B)** and 72 h **(C)**. Effects of different concentrations of omaveloxolone (5, 10, 25, 50, 100, 250, and 500 nM) on the viability of chondrocytes exposed to 10 ng/mL IL-1β for 24 h **(D)**, 48 h **(E)** and 72 h **(F)**. **(G)** The structural formula of omaveloxolone. *p < 0.05 and **p < 0.01 versus the control group. ^#^ *p* < 0.05 and ^##^ *p* < 0.01 versus the IL-1β group (10 ng/ml IL-1β, 0 nM omaveloxolone).

The structural formula of omaveloxolone is presented in Figure 1G. The cell cytotoxicity of different concentrations of omaveloxolone (5, 10, 25, 50, 100, 250, 500, 1000 and 2000 nM) was assessed using a CCK8 assay. No significant cytotoxicity of omaveloxolone on chondrocytes was observed at concentrations below or equal to 500 nM at 24, 48 and 72 h (Figures 1A–C). Therefore, omaveloxolone at concentrations below or equal to 500 nM was used to evaluate the effects on the viability of chondrocytes exposed to IL-1β. The results showed that 10, 25, 50, 100, 250 and 500 nM significantly improved the viability of chondrocytes exposed to IL-1β at 24, 48, and 72 h (Figures 1D–F). 10 nM was the lowest effective concentration able to effectively improve the cell viability and thus was selected for subsequent treatments. At concentration of 25 and 50 nM, omaveloxolone had the best effects on improving the viability of chondrocytes exposed to IL-1β. With the aim to maintain the omaveloxolone therapeutic potential while keeping omaveloxolone concentration as low as possible (to reduce potential adverse effects of drugs on chondrocytes), a concentration of 25 nM was selected for subsequent treatments. Therefore, 10 and 25 nM omaveloxolone were used for the subsequent *in vitro* study based on these results.

### Omaveloxolone suppressed oxidative stress and inflammation in chondrocytes exposed to IL-1β

**FIGURE 2:**
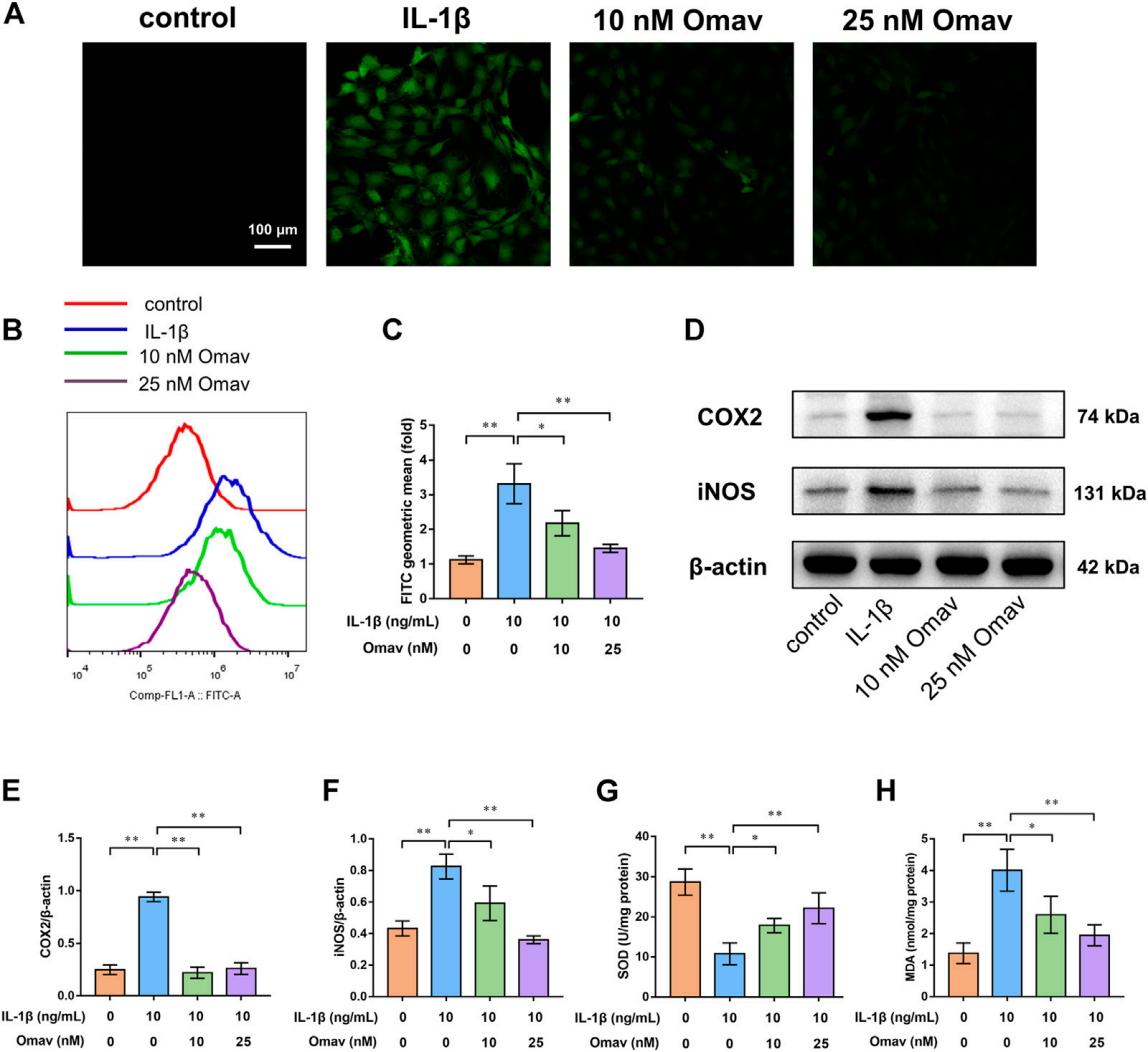
Omaveloxolone exerted antioxidative and anti-inflammatory effects on chondrocytes exposed to IL-1β. **(A)** Representative fluorescence confocal microscopic images of chondrocytes stained with DCFH-DA (scale bar, 100 μm). **(B and C)** Chondrocytes stained with DCFH-DA were assessed by flow cytometry to quantify the ROS level. **(D)** Representative Western blots. Quantitative analysis of COX2 **(E)** and iNOS **(F)** was performed. **(G)** The SOD levels in chondrocytes among the four groups. **(H)** MDA levels in chondrocytes among the four groups. *p < 0.05 and **p < 0.01.

Excessive ROS production causes oxidative stress. DCFH-DA staining was used to evaluate ROS levels in chondrocytes. Representative fluorescence confocal microscopic images are shown in Figure 2A. Green fluorescence indicates ROS production in chondrocytes. Chondrocytes in the control group showed almost no green fluorescence. Bright green fluorescence was observed in IL-1β-treated chondrocytes, whereas the green fluorescence was weak in chondrocytes treated with 10 nM or 25 nM omaveloxolone. In addition, the ROS level was quantified using flow cytometry (Figures 2B,C). The results showed that IL-1β significantly promoted ROS generation in chondrocytes, while 10 and 25 nM omaveloxolone both decreased ROS levels in chondrocytes exposed to IL-1β, suggesting that omaveloxolone effectively suppressed IL-1β-induced ROS generation in chondrocytes.

SOD and MDA are two frequently used indices of oxidative stress. IL-1β significantly increased MAD levels while decreasing SOD levels. Omaveloxolone (10 and 25 nM) reduced MAD levels but increased SOD levels, suggesting that omaveloxolone could prevent IL-1β-induced oxidative stress in chondrocytes (Figures 2G,H).

COX2 and iNOS, two important indicators of inflammation, were measured using Western blotting (Figure 2D). The results showed that IL-1β enhanced the protein expression of COX2 and iNOS, which was partially reversed by administration of 10 and 25 nM omaveloxolone (Figures 2E,F). The results suggested that omaveloxolone could suppress inflammation in chondrocytes exposed to IL-1β.

### Omaveloxolone prevented the apoptosis of chondrocytes exposed to IL-1β

**FIGURE 3:**
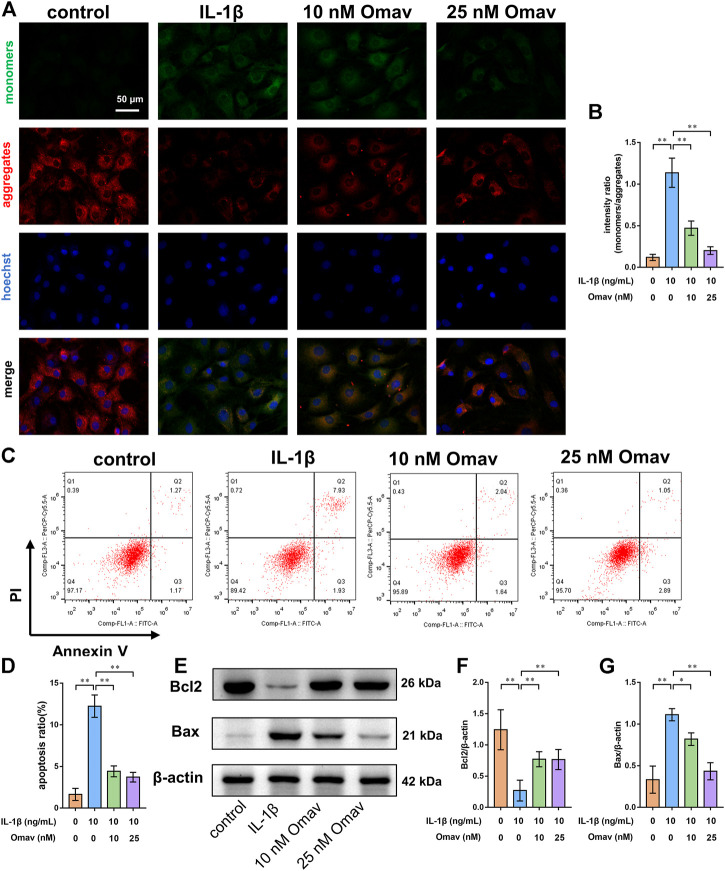
Omaveloxolone prevented IL-1β-induced chondrocyte apoptosis. **(A)** Representative fluorescence confocal microscopic images of chondrocytes stained with JC-1 (scale bar, 50 μm). **(B)** Mitochondrial membrane potential quantitative analysis. **(C)** Chondrocytes were stained with Annexin V/PI and measured by flow cytometry. **(D)** Quantitative analysis of the apoptosis ratio of chondrocytes. **(E)** Representative Western blots. Quantitative analysis of Bcl2 **(F)** and Bax **(G)** was performed. *p < 0.05 and **p < 0.01.

Mitochondrial membrane potential was assessed using JC-1 staining. Representative fluorescence confocal microscopic images are shown in Figure 3A. Green fluorescence indicates decreased mitochondrial membrane potential, while red fluorescence indicates normal mitochondrial membrane potential. The results showed that IL-1β exposure led to decreased mitochondrial membrane potential in chondrocytes, which is a landmark event in early apoptosis. Omaveloxolone (10 and 25 nM) significantly improved the mitochondrial membrane potential in chondrocytes exposed to IL-1β (Figures 3A,B).

Chondrocyte apoptosis was then detected using Annexin V/PI staining. The results of flow cytometry showed that IL-1β increased the apoptosis ratio of chondrocytes, while 10 and 25 nM of omaveloxolone significantly reduced the apoptosis ratio of chondrocytes exposed to IL-1β (Figures 3C,D). In addition, the apoptosis markers Bax and Bcl2 were measured using Western blotting. IL-1β decreased Bcl2 protein levels but increased Bax protein levels in chondrocytes. Omaveloxolone (10 and 25 nM) promoted Bcl2 protein expression and inhibited Bax protein expression in chondrocytes exposed to IL-1β (Figures 3E–G). The results above indicated that omaveloxolone effectively prevented IL-1β-induced apoptosis of chondrocytes.

### Omaveloxolone inhibited ECM degradation of chondrocytes exposed to IL-1β

**FIGURE 4:**
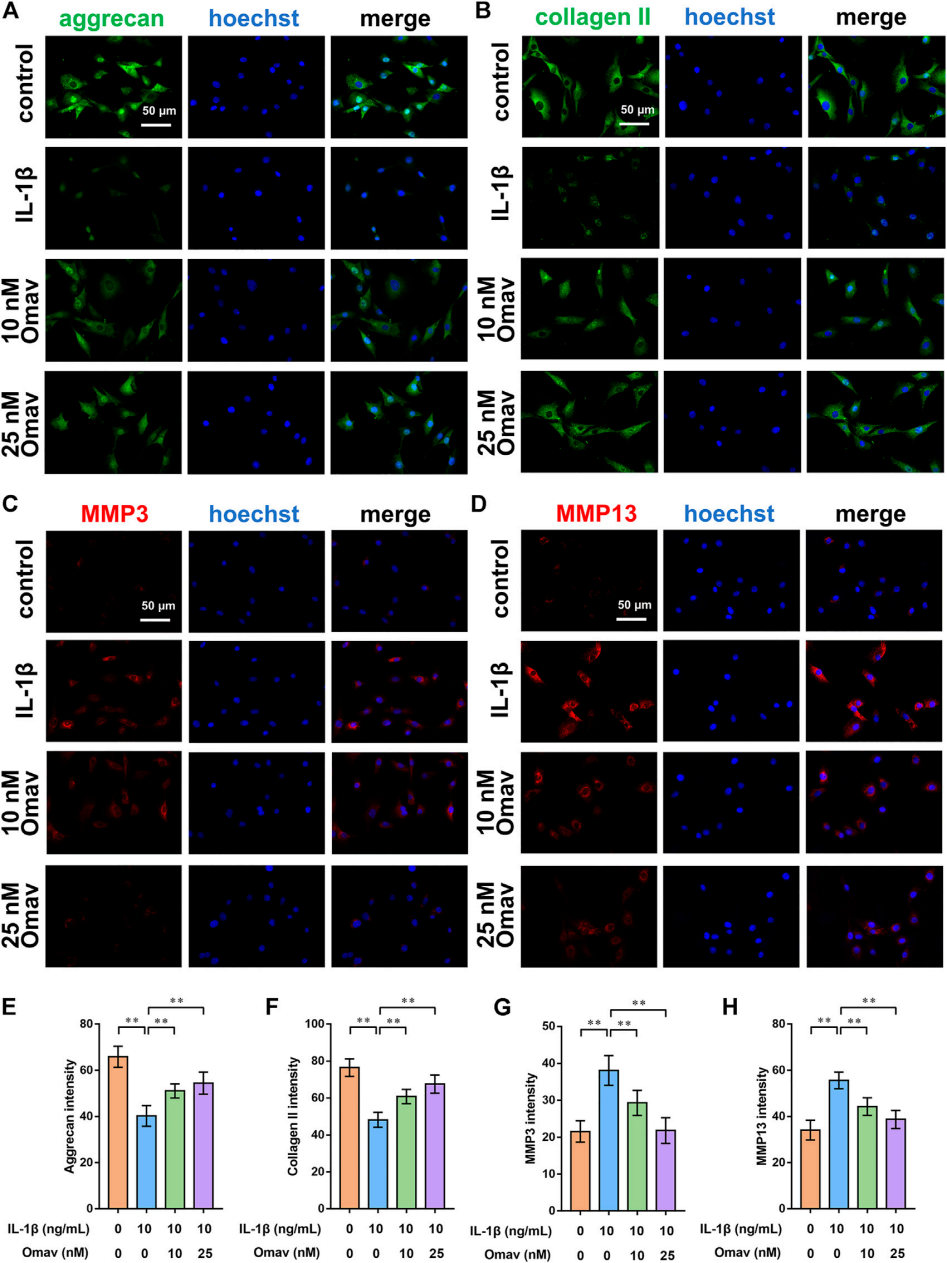
Omaveloxolone suppressed IL-1β-induced ECM degradation *in vitro*. Representative immunofluorescence images of aggrecan **(A)**, collagen type II **(B)**, MMP3 **(C)** and MMP13 **(D)** (scale bar, 50 μm). The results of the quantitative analysis showed that omaveloxolone enhanced aggrecan **(E)** and collagen type II **(F)** protein expression while inhibiting MMP3 **(G)** and MMP13 **(H)** protein expression in chondrocytes exposed to IL-1β. **p < 0.01.

**FIGURE 5:**
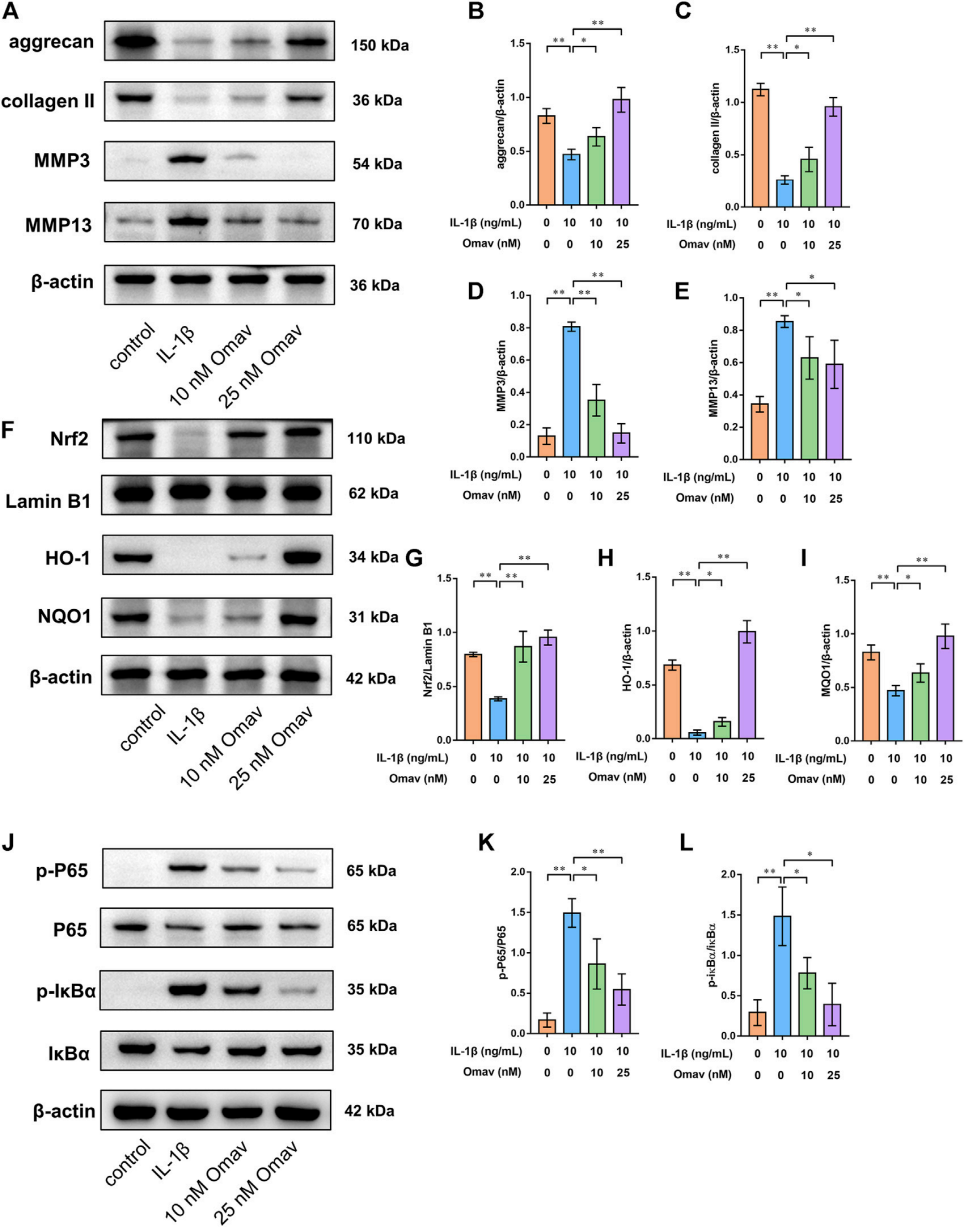
Omaveloxolone activated the Nrf2/ARE signalling pathway while suppressing the NF-κB signalling pathway in chondrocytes exposed to IL-1β. **(A)** Representative Western blots of proteins involved in ECM degradation. Quantitative analysis of aggrecan **(B)**, collagen type II **(C)**, MMP3 **(D)** and MMP13 **(E)** was performed. **(F)** Representative Western blots of proteins involved in the Nrf2/ARE signalling pathway. Quantitative analysis of Nrf2 **(G)**, HO-1 **(H)** and NQO1 **(I)** was performed. **(J)** Representative Western blots of proteins involved in the NF-κB signalling pathway. Omaveloxolone (10 and 25 nM) reduced the p-P65/P65 ratio **(K)** and p-IκBα/IκBα ratio **(L)**. *p < 0.05 and **p < 0.01.

To assess the degree of ECM degeneration, ECM proteins and ECM degrading enzymes, including collagen type II, aggrecan, MMP3 and MMP13, were evaluated using immunofluorescence and Western blotting assays. Representative fluorescence confocal microscopic images are shown in Figures 4A–D. The results of quantitative analysis of the fluorescence intensity showed that IL-1β decreased the expression levels of collagen type II and aggrecan while increasing the expression levels of MMP 3 and MMP 13. Omaveloxolone (10 and 25 nM) increased the expression levels of collagen type II and aggrecan but reduced the expression levels of collagen type II and aggrecan (Figures 4E–H). The results of the Western blotting assay showed that 10 and 25 nM of omaveloxolone significantly promoted collagen type II and aggrecan protein expression while inhibiting MMP 3 and MMP 13 protein expression (Figures 5A–E), which is consistent with the immunofluorescence results. The results indicated that omaveloxolone could inhibit ECM degradation in chondrocytes exposed to IL-1β.

### Omaveloxolone activated the Nrf2/ARE signalling pathway while suppressing the NF-κB signalling pathway in chondrocytes exposed to IL-1β

To explore the underlying mechanism by which omaveloxolone prevents IL-1β-induced apoptosis of chondrocytes, the expression of proteins involved in the Nrf2/ARE and NF-κB signalling pathways was assessed by Western blotting.

To assess the Nrf2/ARE signalling pathway, chondrocytes were pretreated with 10 nM or 25 nM of omaveloxolone for 2 h and then incubated with 10 ng/ml IL-1β for another 24 h. The results showed that 10 and 25 nM of omaveloxolone promoted Nrf2 translocation into the cell nucleus and thus enhanced HO-1 and NQO1 protein expression in chondrocytes exposed to IL-1β, suggesting that the Nrf2/ARE signalling pathway, an important antioxidant signalling pathway, was activated by omaveloxolone (Figures 5F–I).

In addition, for assessment of the NF-κB signalling pathway, chondrocytes were pretreated with 10 nM or 25 nM of omaveloxolone for 2 h and then incubated with 10 ng/ml IL-1β for another 1 h (Xian et al., 2022; Yao et al., 2022). The results of Western blotting analysis showed that 10 and 25 nM of omaveloxolone reduced the p-P65/P65 and p-IκBα/IκBα ratio, indicating that omaveloxolone could suppress the NF-κB signalling pathway in chondrocytes exposed to IL-1β (Figure 5J-L).

### Omaveloxolone alleviated osteoarthritis in rats

**FIGURE 6:**
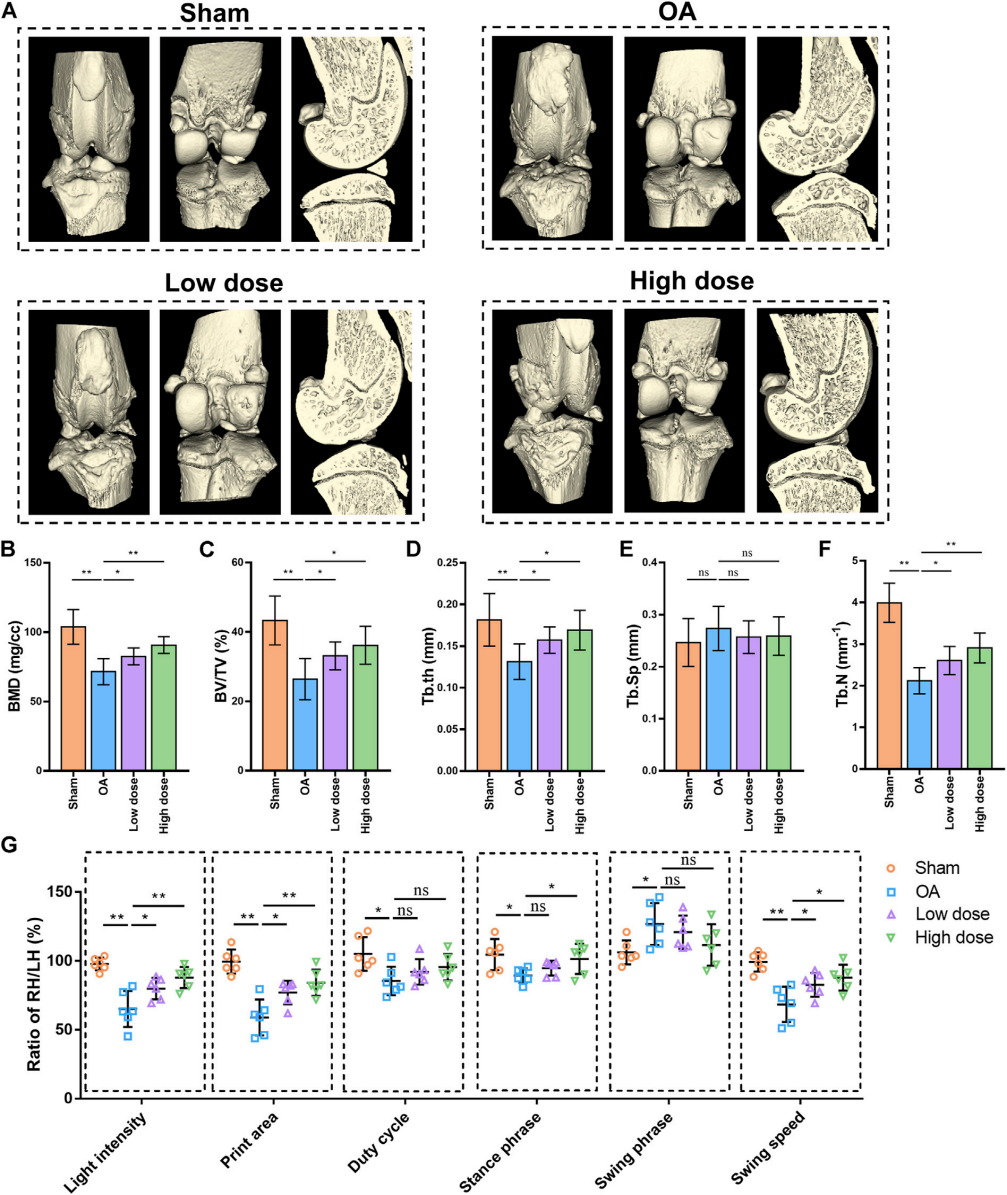
Omaveloxolone relieved pain and suppressed subchondral bone loss and microarchitecture deterioration in OA rats. **(A)** Representative 3D reconstructed images of right knee joints. Quantitative analysis of bone morphometric parameters of tibial subchondral bone, including BMD **(B)**, BV/TV **(C)**, Tb. th **(D)**, Tb. Sp **(E)** and Tb. N **(F)**. **(G)** Gait analysis of light intensity, print area, duty cycle, stance phrase, swing phrase and swing speed. *p < 0.05 and **p < 0.01. n. s., not significant.

Gait analysis is commonly used to assess pain-related behaviours in a rat OA model. CatWalk data were obtained as the right hind (RH)/left hind (LH) limb ratio of light intensity, print area, duty cycle, stance phrase, swing phrase and swing speed. The results of CatWalk analysis showed that the RH/LH ratios of light intensity, print area, duty cycle, stance phrase and swing speed were decreased, while the RH/LH ratio of swing phrase was increased in rats with OA compared with those in control rats. Low dose of omaveloxolone (200 μg/kg) and high dose of omaveloxolone (500 μg/kg) significantly improved the RH/LH ratios of light intensity, print area and swing speed in rats with OA. In addition, high dose of omaveloxolone effectively improved the RH/LH ratios of the stance phase (Figure 6G). These results suggest that omaveloxolone is beneficial for relieving pain in OA rats.

**FIGURE 7:**
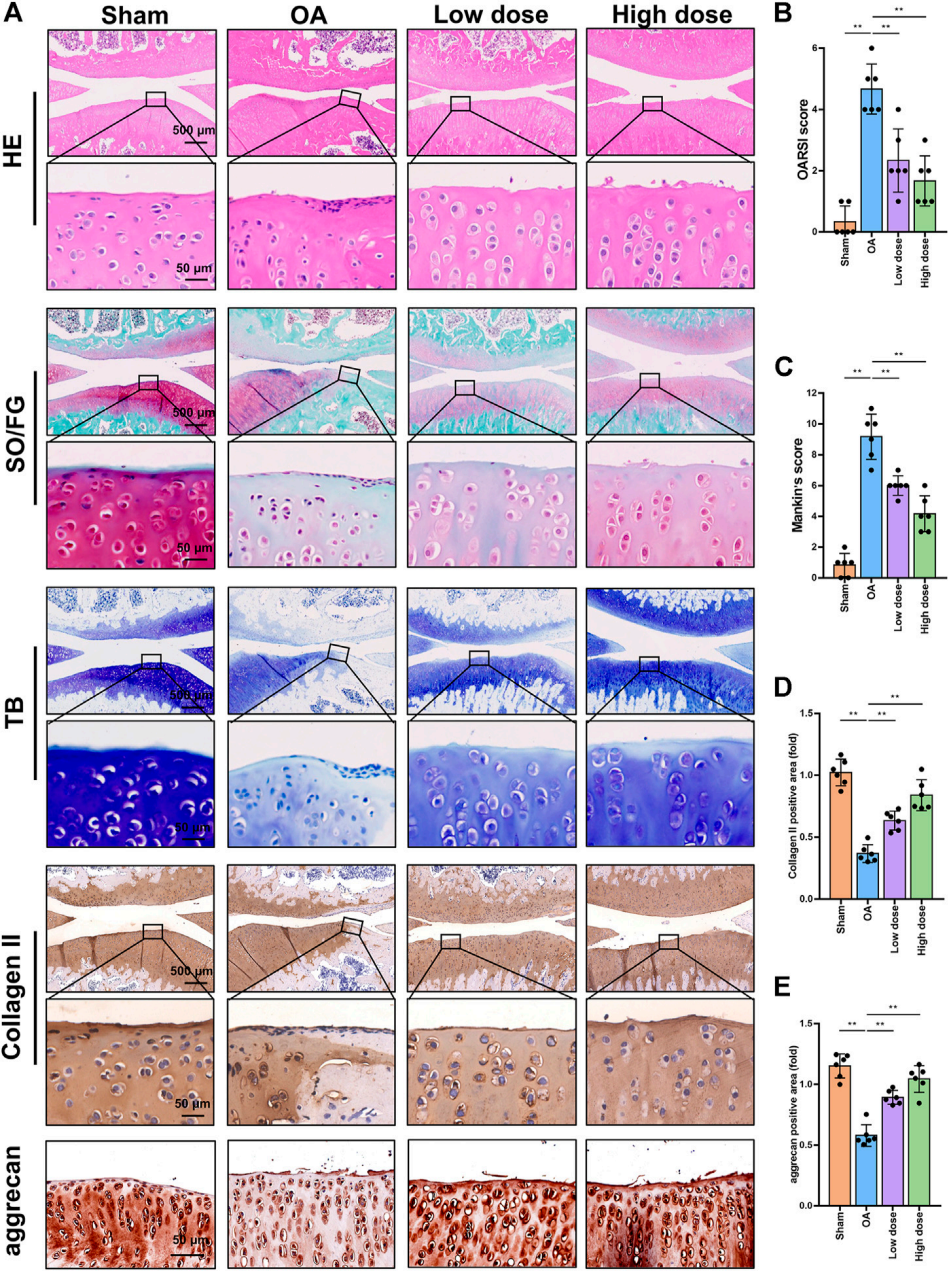
Histopathology and immunohistochemistry analysis of rat knee joints. **(A)** Representative images of HE, SO/FG, TB, collagen type II and aggrecan staining. Images are presented at low magnification (×4 magnification, scale bar, 500 μm) and high magnification (×40 magnification, scale bar, 50 μm). Omaveloxolone (200 and 500 μg/kg) significantly reduced OARSI scores **(B)** and Mankin scores **(C)**. Omaveloxolone (200 and 500 μg/kg) significantly increased collagen type II **(D)** and aggrecan levels **(E)** in OA rats. **p < 0.01.

Histopathology and immunohistochemistry analyses were performed to evaluate the protective effects of omaveloxolone on OA in rats. Representative images of HE, SO/FG and TB staining are shown in Figure 7A. Severe cartilage erosion, massive proteoglycan loss, and decreased number and disordered arrangement of chondrocytes were observed in the OA group. These pathological changes could be ameliorated by low and high doses of oamveloxolone. Histological evaluation was performed using the Osteoarthritis Research Society International (OARSI) scoring system and the modified Mankin scoring system. The results showed that the OARSI scores and Mankin scores of the low-dose and high-dose of oamveloxolone groups were higher than those of the OA groups (Figures 7B,C). Representative immunohistochemistry images of collagen type II and aggrecan are also shown in Figure 7A. The quantitative analysis results showed that.

The expression level of collagen type II in the OA group was lower than that in the control group. Low-dose and high-dose oamveloxolone both increased collagen type II and aggrecan levels *in vivo* (Figures 7D,E).

Furthermore, microCT analysis was performed to assess the changes in the microarchitecture of the subchondral bone. Representative 3D images are shown in Figure 6A. Quantitative analysis of bone microarchitecture parameters showed that both the low dose and high dose of oamveloxolone increased BMD, BV/TV, Tb. th and Tb.N in rats with OA. No significant difference was observed in Tb. Sp among the four groups (Figures 6B–F). The results of behavioural, radiographic, histopathology and immunohistochemistry assays indicate that omaveloxolone effectively alleviated osteoarthritis in rats.

### Omaveloxolone toxicity assessment *in vivo*

**FIGURE 8:**
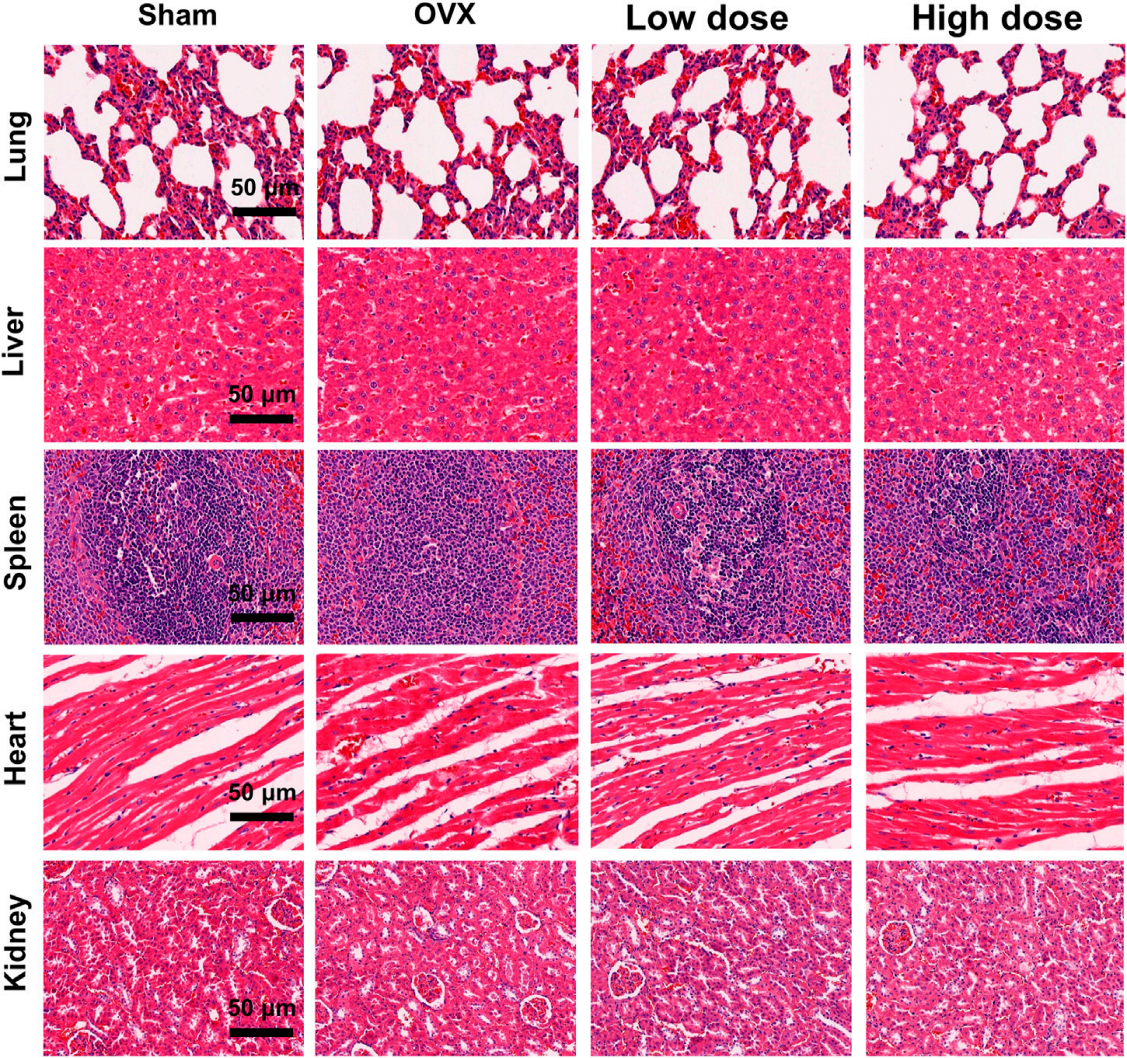
H&E staining of major organs. No obvious pathological changes were observed in the lungs, liver, spleen, heart or kidneys between the four groups, which indicates that 200 and 500 ug/kg omaveloxolone did not cause significant organ toxicity in rats after 8 weeks of treatment. (scale bar, 50 μm).

Major organs (lungs, heart, liver, spleen and kidneys) of rats were harvested and stained with H&E to evaluate the potential toxicity of omaveloxolone. No obvious pathological changes were observed among the four groups, suggesting that 200 and 500 μg/kg omaveloxolone did not cause significant organ toxicity after 8 weeks of treatment. (Figure 8).

## Discussion

Omaveloxolone is a semisynthetic oleanane triterpenoid. Mounting studies have shown that omaveloxolone can exert antioxidative and anti-inflammatory effects via activation of the Nrf2/ARE signalling pathway and inhibition of the NF-κB signalling pathway (Probst et al., 2015; Han et al., 2017; Sun et al., 2020). Therefore, we conducted the current study to explore the effects of omaveloxolone on attenuating OA.

We first explored the effect of omaveloxolone on the viability of chondrocytes exposed to IL-1β *in vitro*. Omaveloxolone at concentrations lower than or equal to 500 nM improved the viability of chondrocytes exposed to IL-1β without significant cytotoxicity. Classic indices correlated with inflammation and oxidative stress were then detected. Omaveloxolone (10 and 25 nM) significantly prevented ROS production, reduced SDO levels and increased MDA levels in chondrocytes treated with IL-1β, suggesting that omaveloxolone could prevent IL-1β-mediated oxidative stress in chondrocytes. In addition, 10 and 25 nM of omaveloxolone effectively suppressed COX2 and iNOS protein expression, indicating that omaveloxolone could inhibit inflammation in chondrocytes. Inflammation and redox imbalance are important pathogenic factors of OA (Henrotin et al., 2003; Lepetsos and Papavassiliou, 2016; Rahmati et al., 2016). Excessive ROS generation and inflammation contribute to the acceleration of chondrocyte apoptosis and cartilage degradation. Therapeutic agents based on antioxidative and anti-inflammatory effects may be readily available and effective (Ziskoven et al., 2010; Zhuo et al., 2012; Tudorachi et al., 2021). This study demonstrated that omaveloxolone protects cells against oxidative injury and inflammation.

Apoptosis is strongly correlated with excessive inflammation and oxidative stress. Excessive chondrocyte apoptosis is considered a key factor for OA progression (Blanco et al., 1998). Therefore, we explored the effects of omaveloxolone on chondrocyte apoptosis. The results showed that omaveloxolone significantly improved mitochondrial membrane potential, enhanced Bcl2 protein expression and inhibited Bax protein expression in chondrocytes exposed to IL-1β. The decrease in mitochondrial membrane potential provided significant evidence of early apoptosis of chondrocytes. Omaveloxolone upregulated the anti-apoptotic protein Bcl2 and downregulated the pro-apoptosis protein Bax, which led to a reduced susceptibility of cells to apoptotic stimuli, such as IL-1β(Dadsena et al., 2021; Lalier et al., 2022; Spitz and Gavathiotis, 2022). The results of the Annexin V/PI staining assay also confirmed that omaveloxolone effectively reduced the apoptosis ratio of chondrocytes treated with IL-1β.

ECM degradation is also a key event in OA progression. The imbalance of synthesis and decomposition of the cartilage matrix caused by various factors results in cartilage degeneration. The results of immunofluorescence and Western blotting assays showed that omaveloxolone significantly promoted collagen type II and aggrecan protein expression while inhibiting MMP 3 and MMP 13 protein expression. Collagen type II and aggrecan are the predominant components in articular cartilage. MMP3 is remarkably active against aggrecan (Vo et al., 2013). The main role of MMP13 is to degrade collagen type II(Akhtar et al., 2017). Cartilage degradation occurs once the homeostasis of these ECM proteins and ECM degrading enzymes is disrupted. Hence, the results demonstrated that omaveloxolone could inhibit ECM degradation in chondrocytes exposed to IL-1β.

**FIGURE 9:**
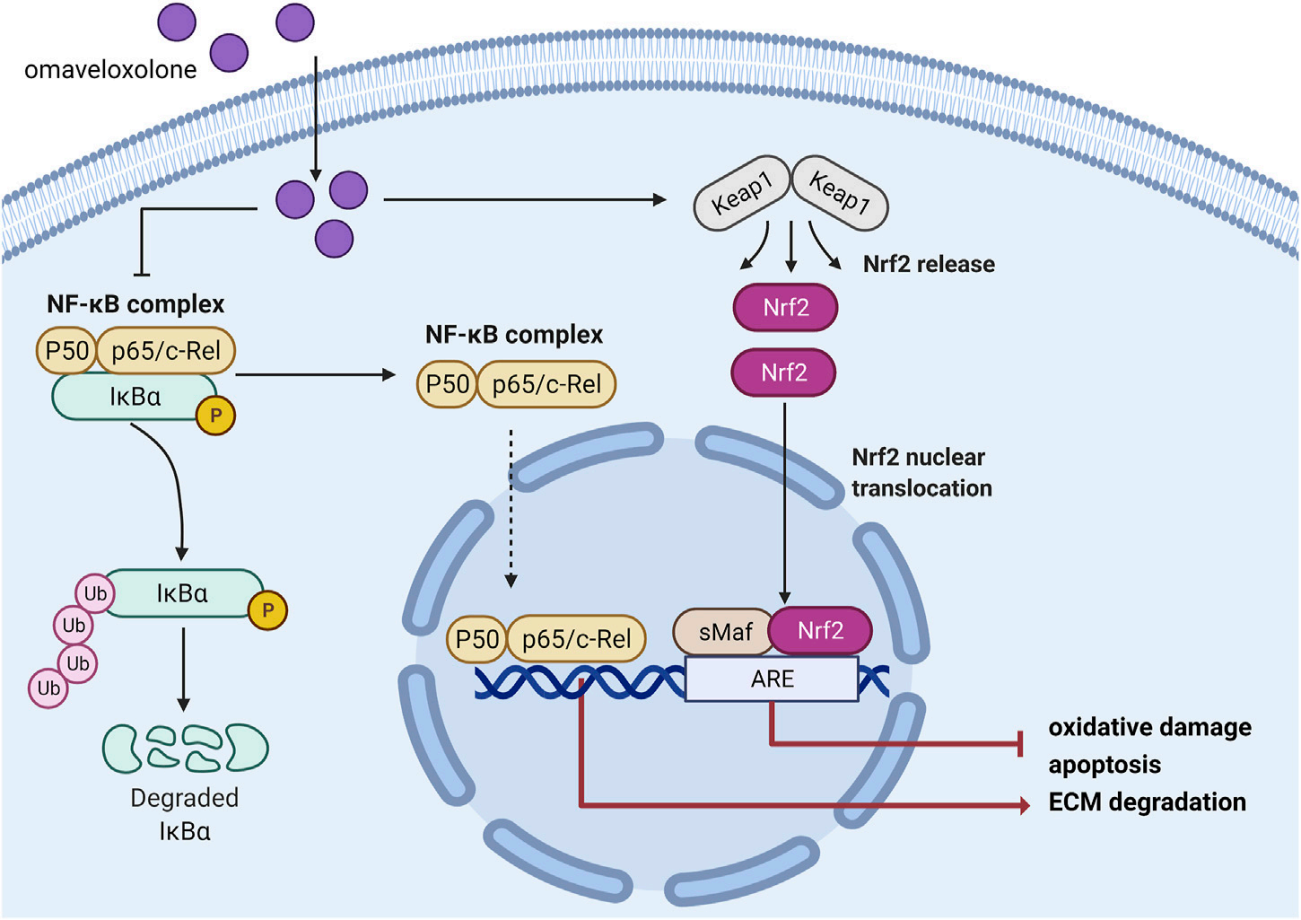
Schematic diagram of the mechanisms by which omaveloxolone affects chondrocytes.

To explore the underlying mechanism by which omaveloxolone prevents IL-1β-induced cell damage, the expression levels of proteins involved in the Nrf2/ARE and NF-κB signalling pathways were assessed. The results showed that omaveloxolone promoted Nrf2 translocation into the cell nucleus, enhanced HO-1 and NQO1 protein expression and reduced the p-P65/P65 and p-IκBα/IκBα ratios in chondrocytes exposed to IL-1β. Nrf2 is a master regulator of antioxidant gene activation. After Nrf2 is translocated into the nucleus it can promote the expression of downstream genes, including HO-1 and NQO1, thereby exerting an antioxidant effect (Khan et al., 2018). Cartilage destruction in Nrf2 knockout OA model mice becomes more obvious and severe, suggesting that Nrf2 activation has chondroprotective potential (Cai et al., 2015). Many drugs have also been shown to protect chondrocytes by activating the Nrf2 signalling pathway. Targeting the Nrf2/ARE signalling pathway might be an effective approach for treating OA (Marchev et al., 2017; Chen et al., 2022). Additionally, the NF-κB signalling pathway is activated in OA joints. The activation of the NF-κB signalling pathway is critical for the expression of inflammation-related proteins in chondrocytes, including COX2, iNOS, MMP3 and MMP13(Marcu et al., 2010). Targeted NF-κB inhibitors have therapeutic potential for the treatment of OA. Therefore, omaveloxolone may exert chondroprotective effects by activating the Nrf2/ARE signalling pathway and suppressing the NF-κB signalling pathway (Figure 9).

A DMM-induced OA rat model was established to examine the effects of omaveloxolone on OA *in vivo*. The results of gait analysis showed that omaveloxolone was beneficial for relieving pain in OA rats. Histopathology and immunohistochemistry analyses provided evidence that omaveloxolone could inhibit chondrocyte loss, improve chondrocyte cell morphology, and increase the levels of proteoglycans, collagen type II and aggrecan in articular cartilage of OA rats. The results of the OARSI scores and Mankin scores also verified the chondroprotective effect of omaveloxolone *in vivo*.

The results of the microCT analysis suggest that omaveloxolone can effectively suppress subchondral bone loss and microarchitecture deterioration in OA rats. These findings support the therapeutic potential of omaveloxolone for OA treatment. Considering that the systemic administration of omaveloxolone may cause potential organ toxicity, H&E staining of the lungs, heart, liver, spleen and kidneys was performed, and the results suggest an absence of major organ toxicity, which provides preliminary evidence of the safety of omaveloxolone in rats.

In conclusion, the present study indicates that omaveloxolone can exert antioxidative, anti-inflammatory, antiapoptotic and anti-ECM degradation effects via activation of the Nrf2/ARE signalling pathway and inhibition of the NF-κB signalling pathway in chondrocytes *in vitro.* Moreover, omaveloxolone attenuates OA progression *in vivo.* However, our study does have some limitations. First, only one time point was investigated in our *in vivo* study. Therefore, data on the long-term effects of omaveloxolone in the treatment of OA and assessments of the potential toxicity of long-term administration of omaveloxolone are lacking. In addition, further studies are needed to explore other possible underlying molecular mechanisms of omaveloxolone in chondrocyte protection.

## Data Availability

The original contributions presented in the study are included in the article/supplementary materials, further inquiries can be directed to the corresponding authors.
